# Impact of Comorbid Personality Disorder on the Risk of Involuntary Hospitalization in Patients Referred for Urgent Forensic Assessment: A Cross-Sectional Study

**DOI:** 10.3390/brainsci14100961

**Published:** 2024-09-25

**Authors:** Axel Dossa, Matthieu Hein, Oussama Bikrani, Benjamin Wacquier, Camille Point

**Affiliations:** 1 Faculté de Médecine, Université Libre de Bruxelles (ULB), 1070 Bruxelles, Belgium; axel.dossa@ulb.be (A.D.); oussama.bikrani@ulb.be (O.B.); 2 Service de Psychiatrie et Laboratoire du Sommeil, Hôpital Universitaire de Bruxelles, Université Libre de Bruxelles (ULB), 1070 Bruxelles, Belgium; benjamin.wacquier@hubruxelles.be (B.W.); camille.point@hubruxelles.be (C.P.); 3 Laboratoire de Psychologie Médicale et Addictologie (ULB312), Université Libre de Bruxelles (ULB), 1020 Bruxelles, Belgium

**Keywords:** personality disorder, involuntary psychiatric hospitalization, forensic assessment, psychiatric emergency, severe psychiatric disorders

## Abstract

Background/Objectives: In Belgium, involuntary psychiatric hospitalization is authorized in the presence of certain criteria governed by the law relating to the protection of the mentally ill. The number of involuntary hospitalizations has been increasing continuously in recent years. Since personality disorders are frequent comorbidities in involuntarily hospitalized patients, the aim of this study was to investigate the potential role played by comorbid personality disorders in the decisions about involuntary hospitalization made during urgent forensic assessment. Methods: A total of 565 individuals were retrospectively recruited from the database of urgent forensic assessment carried out in the Psychiatric Emergency Department. Logistic regression analyses were performed to investigate the risk of involuntary hospitalization associated with comorbid personality disorders in patients referred for urgent forensic assessment. Results: 66.7% of urgent forensic assessments resulted in involuntary hospitalization. In addition, comorbid personality disorders (especially borderline personality disorder) were associated with a lower risk of involuntary hospitalization in patients referred for urgent forensic assessment. Conclusions: In this study, we demonstrated that urgent forensic assessments frequently result in involuntary hospitalizations. Furthermore, this study highlighted that comorbid personality disorders (especially borderline personality disorder) appeared to have a major impact on the decision not to involuntarily hospitalize patients referred for urgent forensic assessment. These elements therefore justify the establishment of adequate clinical reflection to avoid the stigmatization related to these frequent comorbidities in patients at risk of involuntary hospitalization.

## 1. Introduction

In Belgium, involuntary psychiatric hospitalizations are authorized according to the criteria of the law relating to the protection of the mentally ill of 26 June 1990 [1,2]: (1) presence of an acute psychiatric episode, (2) presence of serious danger for oneself or others, and (3) refusal of appropriate care.

The absence of an adequate therapeutic alternative must also be considered when making decisions. In practice, two procedures exist to apply this law: (1) the ordinary procedure, initiated by a request by the justice of the peace, according to article 5, and (2) the emergency procedure, initiated by a request to the King’s Prosecutor, according to article 9.

As part of the emergency procedure, and if the latter takes place in the Brussels Capital region, the psychiatric assessment aimed at evaluating the criteria of the law is carried out within one of the four approved emergency services. This is the Nixon procedure, unique to the Brussels Capital region, which allows for a fair distribution of the total evaluations between approved services. This procedure promotes the active search for alternatives to compulsory hospitalization in order to restrict the use of involuntary psychiatric hospitalizations to situations requiring this type of measure.

If an assessment proves positive, that is to say if the three legal criteria described above are present concomitantly, involuntary psychiatric hospitalization in an approved secured unit is organized and the patient is said to be “placed under observation”. If an assessment proves negative (in the absence of all three legal criteria), the patient is free to leave the hospital and to pursue care of his or her choice. The evaluation performed by the psychiatrist in the emergency department is validated by the approval of the king’s prosecutor.

In Europe and throughout the world [3,4], involuntary hospitalizations for psychiatric reasons follow different legal and organizational criteria. However, we observe a consensus around the implementation of measures to reduce coercion in psychiatry on the international scene [5,6]. Despite this observation, in Belgium, it has been noted that there has been a constant increase over time in the number of involuntary psychiatric hospitalizations [7,8] and forensic assessments carried out in emergency departments. According to a report from the federal public health service, in Belgium, in 2021, 9.8% of psychiatric hospitalizations are imposed by a legal measure. Among the latter, 69.3% are involuntary hospitalizations, the majority of which take place in the region of Brussels-Capitale [8]. This report highlights an increase in the number of involuntary psychiatric hospitalizations of 30.1% between 2012 and 2021 [8]. However, according to a 2021 report, the diagnosis of personality disorder, particularly cluster B, only represents 2.7% of involuntary psychiatric hospitalizations that year [8].

In the literature, numerous risk factors favoring involuntary psychiatric hospitalizations have been identified over time [9,10,11,12]: a precarious socio-economic situation, male gender, psychiatric diagnosis, immigration status, the psychiatrist who carries out the forensic assessment, the absence of setting up outpatient monitoring, etc. In the same way, some factors have been identified as worsening the prognosis of clinical evolution of patients and/or prolonging the duration of hospitalization. Among these problematic factors, we find the presence of comorbid personality disorders [13,14,15] in patients presenting a psychiatric disorder as defined by the DSM 5 [16].

In this context, it is essential to study the factors favoring involuntary hospitalizations in patients referred as psychiatric emergencies in the context of the urgent procedure as described by the law of 26 June 1990 in order to open up new perspectives for prevention and care in this particular subpopulation. The objective of this retrospective study was therefore to investigate the potential role played by comorbid personality disorders in the decision of involuntary psychiatric hospitalization during urgent forensic assessment.

## 2. Materials and Methods

### 2.1. Population

565 adult patients were retrospectively selected from the database of the Psychiatric Emergency Department of the Brussels University Hospital which contains urgent forensic assessments carried out between January 2021 and December 2023.

The inclusion criteria were: age ≥ 18 years and admission to the Psychiatric Emergency Department for an urgent forensic assessment.

The exclusion criteria were: age < 18 years, presence of incomplete data concerning urgent forensic assessment, and admission to the Psychiatric Emergency Department for a voluntary outpatient consultation.

Finally, for each patient included, only the first assessment available over the period studied was selected.

### 2.2. Patient Assessment during the Stay in the Psychiatric Emergency Department

Patients referred for urgent forensic assessment are escorted by police forces during their admission to the Emergency Department of the Brussels University Hospital. Indeed, patients are deprived of their freedoms during the psychiatric assessment at the request of the King’s Prosecutor within the framework of the strict application of the law. After their admission, all these patients underwent an assessment of their vital parameters by nursing staff and a somatic assessment by an emergency physician to exclude an organic cause for their clinical condition. Subsequently, a systematic psychiatric interview is carried out by a junior psychiatrist to confirm or deny the presence of the legal criteria necessary for the establishment of involuntary psychiatric hospitalization. Following this systematic psychiatric interview and supervision by a senior psychiatrist, a decision regarding the establishment of involuntary psychiatric hospitalization may be taken immediately or postponed for 24 h to allow a clinical reassessment of the situation in the event of a patient who cannot be assessed directly, who is intoxicated (medications, alcohol or drugs), or who needs complementary exams (head CT, evaluation by another specialist, etc.). If the urgent forensic assessment is negative due to the absence of legal criteria, the patient is referred for voluntary hospitalization or outpatient psychiatric care. If the urgent forensic assessment is rendered positive by the presence of all the legal criteria, patients are then placed in isolation cells or under restraint after medication to await their transfer to a hospital approved for involuntary psychiatric hospitalizations.

### 2.3. Statistical Analyses

Stata software version 14 was used to perform the statistical analyses. Continuous variables were described by their median (P25–P75) and Wilcoxon tests were used for comparison analyses, whereas categorical variables were described by percentages and Chi-square tests were used for comparison analyses. In order to enable these descriptive and comparative analyses, our patient sample was divided into a group with negative urgent forensic assessment and a group with positive urgent forensic assessment.

The risk of involuntary psychiatric hospitalization associated with comorbid personality disorders and potential confounding factors was investigated using univariate logistic regression analyses. After a review of the literature by the authors of this study on main factors associated with involuntary psychiatric hospitalizations [9,10,11,12,17,18,19], the confounding factors included in these univariate analyses were the following: age (categorized as <30 years, ≥30 years), socioeconomic status of the residential municipality (categorized as low, intermediate, high), psychological/psychiatric follow-up (categorized as no, past, current), previous hospitalizations (categorized as no, voluntary, involuntary, voluntary + involuntary), mood disorder (categorized as no, unipolar depressive disorder, bipolar disorder) and, as binary variables, gender, marital status, current general practitioner, medical contact within 3 months before assessment, assessment initiated by health professional, somatic comorbidities on admission, antidepressant treatment on admission, benzodiazepines on admission, neuroleptic on admission, thymostabilizer on admission, other psychotropic drugs on admission, third party contacted during the assessment, placed in isolation cell, psychotic disorder, suicidality, substance use disorder, anxiety disorder, and major behavioral disorder. Regarding the cut-off for age, it was selected based on the available literature indicating that young adults are at increased risk of involuntary psychiatric hospitalizations [18].

Only confounding factors significant in univariate analyses were included hierarchically in the multivariate logistic regression models in order to adjust the risk of involuntary psychiatric hospitalization associated with comorbid personality disorders.

For the final model, the adequacy was verified by the Hosmer and Lemeshow test whereas the specificity was verified by the Link test.

Results were considered significant when the *p*-value was <0.05.

## 3. Results

### 3.1. Univariate Analyses (Table 1)

In our sample, 66.7% of urgent forensic assessments were positive, leading to involuntary psychiatric hospitalization. Female gender, being single, current or past psychiatric/psychological follow-up, history of voluntary or involuntary hospitalization, presence of a medical contact in the previous 3 months, a procedure initiated by a health professional, the use of neuroleptics on admission, the use of mood stabilizers on admission, the need to be placed in an isolation cell, the presence of a bipolar disorder, the presence of a psychotic disorder, the presence of suicidality, the absence of a substance use disorder, the absence of a personality disorder, and the presence of a behavioral disorder were associated with higher risk of involuntary psychiatric hospitalization in patients referred for urgent forensic assessment. Finally, the prevalence of comorbid personality disorders was 14.2% (1.8% cluster A, 11.7% cluster B [3.5% antisocial personality and 8.2% borderline personality] and 0.7% cluster C) in our sample of patients referred for urgent forensic assessment.

**Table 1:** Univariate analyses (*n* = 565).

Variables	Categories	%	Assessment Negative	Assessment Positive	*p*-Value Chi^2^	OR (CI 95%)	*p*-Value
Gender	Female (*n* = 243)	43.0%	35.6%	46.7%	0.012	1	0.013
	Male (*n* = 322)	57.0%	64.4%	53.3%		0.63 (0.44 to 0.91)	
Age (years)	<30 (*n* = 166)	29.4%	29.8%	29.2%	0.881	1	0.881
	≥30 (*n* = 399)	70.6%	70.2%	70.8%		1.03 (0.70 to 1.51)	
Single	No (*n* = 141)	25.0%	30.3%	22.3%	0.037	1	0.038
	Yes (*n* = 424)	75.0%	69.7%	77.7%		1.52 (1.02 to 2.25)	
Socioeconomic status of the residential municipality	Low (*n* = 188)	33.3%	36.2%	31.8%	0.511	1	0.511
	Intermediate (*n* = 260)	46.0%	45.2%	46.4%		1.17 (0.79 to 1.73)	
	High (*n* = 117)	20.7%	18.6%	21.8%		1.33 (0.81 to 2.18)	
Psychological/psychiatric follow-up	No (*n* = 229)	40.5%	52.7%	34.5%	<0.001	1	<0.001
	Past (*n* = 132)	23.4%	18.6%	25.7%		2.11 (1.32 to 3.37)	
	Current (*n* = 204)	36.1%	28.7%	39.8%		2.12 (1.41 to 3.18)	
Previous hospitalizations	No (*n* = 307)	54.3%	68.0%	47.5%	<0.001	1	<0.001
	Voluntary (*n* = 92)	16.3%	12.8%	18.0%		2.03 (1.21 to 3.40)	
	Forced (*n* = 101)	17.9%	9.6%	22.0%		3.30 (1.89 to 5.76)	
	Voluntary + forced (*n* = 65)	11.5%	9.6%	12.5%		1.87 (1.04 to 3.36)	
Current general practitioner	No (*n* = 370)	65.5%	66.5%	65.0%	0.723	1	0.723
	Yes (*n* = 195)	34.5%	33.5%	35.0%		1.07 (0.74 to 1.55)	
Medical contact within 3 months before assessment	No (*n* = 329)	58.2%	65.4%	54.6%	0.014	1	0.015
	Yes (*n* = 236)	41.8%	34.6%	45.4%		1.57 (1.09 to 2.26)	
Assessment initiated by health professional	No (*n* = 296)	52.4%	64.9%	46.2%	<0.001	1	<0.001
	Yes (*n* = 269)	47.6%	35.1%	53.8%		2.16 (1.50 to 3.10)	
Somatic comorbidities on admission	No (*n* = 395)	69.9%	68.6%	70.6%	0.636	1	0.636
	Yes (*n* = 170)	30.1%	31.4%	29.4%		0.91 (0.62 to 1.33)	
Antidepressant treatment on admission	No (*n* = 443)	78.4%	76.6%	79.3%	0.460	1	0.460
	Yes (*n* = 122)	21.6%	23.4%	20.7%		0.85 (0.56 to 1.30)	
Benzodiazepines on admission	No (*n* = 431)	76.3%	78.2%	75.3%	0.451	1	0.452
	Yes (*n* = 134)	23.7%	21.8%	24.7%		1.17 (0.77 to 1.78)	
Neuroleptic on admission	No (*n* = 399)	70.6%	77.1%	67.4%	0.016	1	0.017
	Yes (*n* = 166)	29.4%	22.9%	32.6%		1.63 (1.09 to 2.44)	
Thymostabilizer on admission	No (*n* = 531)	94.0%	97.9%	92.0%	0.006	1	0.011
	Yes (*n* = 34)	6.0%	2.1%	8.0%		3.98 (1.38 to 11.46)	
Other psychotropic drugs on admission	No (*n* = 503)	89.0%	88.3%	89.4%	0.696	1	0.696
	Yes (*n* = 62)	11.0%	11.7%	10.6%		0.90 (0.52 to 1.56)	
Third party contacted during the assessment	No (*n* = 264)	46.7%	49.5%	45.4%	0.356	1	0.356
	Yes (*n* = 301)	53.3%	50.5%	54.6%		1.18 (0.83 to 1.67)	
Placed in isolation cell	No (*n* = 199)	35.2%	72.9%	16.5%	<0.001	1	<0.001
	Yes (*n* = 366)	64.8%	27.1%	83.5%		13.65 (8.96 to 20.80)	
Mood disorder	No (*n* = 350)	62.0%	68.1%	58.9%	<0.001	1	<0.001
	Unipolar depressive disorder (*n* = 143)	25.3%	27.1%	24.4%		1.04 (0.69 to 1.56)	
	Bipolar disorder (*n* = 72)	12.7%	4.8%	16.7%		4.04 (1.94 to 8.39)	
Psychotic disorder	No (*n* = 249)	44.1%	67.5%	32.4%	<0.001	1	<0.001
	Yes (*n* = 316)	55.9%	32.5%	67.6%		4.35 (2.99 to 6.32)	
Suicidality	No (*n* = 400)	70.8%	76.6%	67.9%	0.032	1	0.033
	Yes (*n* = 165)	29.2%	23.4%	32.1%		1.55 (1.04 to 2.31)	
Substance use disorder	No (*n* = 342)	60.5%	46.8%	67.4%	<0.001	1	<0.001
	Yes (*n* = 223)	39.5%	53.2%	32.6%		0.43 (0.30 to 0.61)	
Anxiety disorder	No (*n* = 471)	83.4%	86.2%	82.0%	0.206	1	0.207
	Yes (*n* = 94)	16.6%	13.8%	18.0%		1.37 (0.84 to 2.24)	
Comorbid personality disorder	No (*n* = 485)	85.8%	76.6%	90.5%	<0.001	1	<0.001
	Yes (*n* = 80)	14.2%	23.4%	9.5%		0.35 (0.21 to 0.56)	
Major behavioral disorder	No (*n* = 474)	83.9%	88.8%	81.4%	0.024	1	0.026
	Yes (*n* = 91)	16.1%	11.2%	18.6%		1.81 (1.07 to 3.06)	
Assessment positive	No (*n* = 188)	33.3%					
	Yes (*n* = 377)	66.7%					
	Median (P25-P75)				Wilcoxon test		
Age (years)	37 (28–48)		38 (28–48)	37 (28–49)	0.942		

### 3.2. Multivariate Analyses (Table 2)

After adjusting for the main significant confounding factors during univariate analyses, multivariate logistic regression analyses demonstrated that comorbid personality disorders were associated with lower risk of involuntary psychiatric hospitalization during an urgent forensic assessment.

**Table 2:** Multivariate analyses (*n* = 565).

Variables	Model OR Adjusted (CI 95%)	*p*-Value
Personality disorder		0.003
No	1	
Yes	0.33 (0.16 to 0.67)	

Model adjusted for gender, marital status, psychological/psychiatric follow-up, previous hospitalizations, medical contact within 3 months before assessment, initiator of assessment, neuroleptic on admission, thymostabilizer on admission, placed in isolation cell, mood disorder, psychotic disorder, suicidality, substance use disorder and major behavioral disorder.

### 3.3. Additional Multivariate Analyses for Personality Clusters (Table 3)

After adjustment for the main confounding factors identified during the univariate analyses, additional multivariate logistic regression analyses revealed that unlike other clusters of comorbid personality disorders, only cluster B of comorbid personality disorders was associated with lower risk of involuntary psychiatric hospitalization during an urgent forensic assessment.

**Table 3:** Additional multivariate analyses for personality clusters (*n* = 565).

Variables	%	Assessment Negative	Assessment Positive	Model 1 OR Unadjusted (CI 95%)	*p*-Value	Model 2 OR Adjusted (CI 95%)	*p*-Value
Personality disorder					<0.001		0.008
No	85.8% (*n* = 485)	76.6%	90.5%	1		1	
Cluster B	11.7% (*n* = 66)	19.7%	7.7%	0.33 (0.20 to 0.56)		0.30 (0.14 to 0.64)	
Other clusters	2.5% (*n* = 14)	3.7%	1.8%	0.42 (0.15 to 1.23)		0.59 (0.09 to 3.89)	

Model 1 = Model unadjusted. Model 2 = Model adjusted for gender, marital status, psychological/psychiatric follow-up, previous hospitalizations, medical contact within 3 months before assessment, initiator of assessment, neuroleptic on admission, thymostabilizer on admission, placed in isolation cell, mood disorder, psychotic disorder, suicidality, substance use disorder and major behavioral disorder.

### 3.4. Additional Multivariate Analyses for Cluster B Personality Subtypes (Table 4)

After adjustment for the main confounding factors identified during the univariate analyses, additional multivariate logistic regression analyses revealed that unlike other clusters of comorbid personality disorders and cluster B—Antisocial personality, only cluster B—Borderline personality was associated with lower risk of involuntary psychiatric hospitalization during an urgent forensic assessment.

**Table 4:** Additional multivariate analyses for cluster B personality subtypes (*n* = 565).

Variables	%	Assessment Negative	Assessment Positive	Model 1 OR Unadjusted (CI 95%)	*p*-Value	Model 2 OR Adjusted (CI 95%)	*p*-Value
Personality disorder					<0.001		0.013
No	85.8% (*n* = 485)	76.6%	90.5%	1		1	
Cluster B—Antisocial personality	3.5% (*n* = 20)	6.9%	1.9%	0.23 (0.09 to 0.58)		0.55 (0.15 to 2.08)	
Cluster B—Borderline personality	8.2% (*n* = 46)	12.8%	5.8%	0.39 (0.21 to 0.71)		0.23 (0.09 to 0.56)	
Other clusters	2.5% (*n* = 14)	3.7%	1.8%	0.42 (0.15 to 1.23)		0.59 (0.09 to 3.92)	

Model 1 = Model unadjusted. Model 2 = Model adjusted for gender, marital status, psychological/psychiatric follow-up, previous hospitalizations, medical contact within 3 months before assessment, initiator of assessment, neuroleptic on admission, thymostabilizer on admission, placed in isolation cell, mood disorder, psychotic disorder, suicidality, substance use disorder and major behavioral disorder.

## 4. Discussion

The key finding of this study is that the presence of a comorbid personality disorder is associated with a reduced likelihood of involuntary psychiatric hospitalization under Belgian legal criteria. The literature corroborates the fact that the majority of patients with a personality disorder are not hospitalized, whatever the mode of hospitalization [20]. Indeed, similar to our study where only 9.5% of involuntarily hospitalized patients presented comorbid personality disorders, an Italian study conducted over 5 years highlighted a prevalence of 11.4% of comorbid personality disorders in this particular subpopulation [21]. However, although comorbid personality disorders appear to be initially associated with a lower risk of both voluntary and involuntary hospitalization, this comorbidity is frequent (14–20%) in patients hospitalized in psychiatry, which underlines the importance of this problem for hospital psychiatrists [21]. On the other hand, the potential impact of comorbid personality disorders on the risk of involuntary psychiatric hospitalization appears to be related to the severity of the underlying psychiatric disorder, which could be due to the presence of an increased risk to themselves or to others in the event of co-occurrence of these comorbidities with a severe psychiatric disorder [22]. However, these potentially contradictory results with those of our study carried out in Belgium could be explained by different legal criteria for involuntary psychiatric hospitalization in the United Kingdom with greater weight given to the criterion of dangerousness [22]. Finally, consistent with the literature [9,20,21], we confirmed that comorbid personality disorders are more frequent (23.4%) in patients who do not meet the criteria for involuntary hospitalization, which could be explained by the fact that the patients hospitalized on a voluntary basis and those hospitalized under compulsion correspond to different populations, with different psychiatric profiles and mental health care needs [21].

Another factor to consider when interpreting this lower risk of involuntary psychiatric hospitalization in cases of comorbid personality disorders demonstrated in our study is the potential impact of country-specific legal specificity. Indeed, the literature describes different types of care and constraints depending on the concerned country, the law in place and the organization of the healthcare system. In some countries, personality disorders are not considered a mental illness, and patients are therefore generally not hospitalized [23]. When they are, hospitalizations are short-term and only on a voluntary basis [23]. In this context, hospitalizations occur during an acute crisis, with imminent danger, and have the sole objective of stabilizing the current situation. In these countries, involuntary psychiatric hospitalization is reserved for patients with a psychotic disorder and/or an immediate risk of suicide [23]. However, in other countries, the presence of a personality disorder is a sufficient reason for short hospitalization without consent, solely with the aim of preventing any self-aggressive act [24]. Suicidal risk is an important element in the decision to involuntarily hospitalize. Indeed, the concomitant presence of a suicidal risk and a personality disorder significantly increases the risk of involuntary psychiatric hospitalization [20]. The fact that many psychiatrists believe that hospitalization without consent, except in the presence of a high suicidal risk, may be useless, or even have a pejorative effect on the mental state of the patient, seems to be the main hypothesis explaining this result [20].

Involuntary psychiatric hospitalizations are longer than voluntary ones, probably due to poor patient compliance or the severity of the pathology [21]. The presence of a comorbid personality disorder favors an increase in the duration of hospitalization compared to the absence of a personality disorder [22]. Some studies specify that depending on the personality disorder cluster, the duration of hospitalization may vary [25]. Among the personality types, borderline personalities most frequently request hospitalization and psychiatric care in general, whereas cluster C personalities require less care and are less represented in the samples [26]. Generally speaking, borderline, antisocial and schizotypal personalities are the most represented in psychiatric units, whether the hospitalization is forced or voluntary [25]. In addition, borderline personalities are associated with shorter hospitalizations, but also with a higher frequency of hospitalization compared to the absence of borderline personality disorder [25,27]. The presence of a borderline personality is furthermore regularly associated with dangerous situations for the patient or for others, leading to urgent treatment [25,28]. The latter are more serious, involving medication and restraint, but also increased vigilance by the care teams in order to avoid self- or hetero-aggressive acts [27,29].

Despite these numerous data in favor of a negative impact of comorbid personality disorders (especially borderline personality disorder) on the prognosis of psychiatric patients, some elements could help to better understand the potentially counter-intuitive results of our study characterized by a protective effect of these comorbidities on the risk of involuntary psychiatric hospitalization. Indeed, some studies have shown that some personality disorders are correlated with a negative perception of the patient by some health professionals [30,31,32,33]. Indeed, these patients are often perceived as being less inclined to good therapeutic compliance but also as being complicated patients to take care of, in particular because of the impact of the pathology on their social relationships [30,31,33]. These negative attitudes on the part of the care staff may lead to several consequences, namely poorer care quality, a lack of empathy, little confidence in the effectiveness of treatment, and a reduction in the well-being of caregivers. These difficulties are more present in borderline personality compared to other personality types [30,32]. Patients with a personality disorder are perceived as being responsible for their actions and their behaviors judged to be deliberate. As a result, they provoke, in reaction, negative feelings in their interlocutor, which can lead to discriminatory behavior in therapeutic choices, or even an underestimation of the seriousness of the patient’s psychiatric situation. This could be one of the reasons explaining the low rate of positive urgent forensic assessment when comorbid personality disorders (especially borderline personality disorder) are present. However, it is important to note that in these patients with comorbid personality disorders, these different problematic behaviors favoring their negative perception by healthcare professionals may be linked to both functional and structural brain abnormalities [34]. Indeed, it has been demonstrated that in patients with comorbid personality disorders (particularly borderline personality disorder), neural circuits involved in the regulation of emotions and in some cognitive processes showed alterations in the processing of information [35,36]. Furthermore, in these patients, the presence of alterations in some brain structures seems to promote the occurrence of some behavioral disorders (impulsivity, aggression and suicidality) [37,38]. Thus, the occurrence of problematic behaviors that promote negative perceptions by healthcare professionals of patients with personality disorders does not appear to be solely due to deliberate choices on their part [39]. Finally, it was demonstrated that the development of training for healthcare professionals on this neurobiological reality of problematic behaviors in patients with comorbid personality disorders helped to reduce their negative perception of this particular subgroup of patients [40].

When the assessment is negative for patients with a comorbid personality disorder, it is important to question their future. Several factors must then be taken into account. Indeed, socio-economic status and ethnic origin are major obstacles to accessing mental health care [41,42]. Two options are available to these patients: outpatient follow-up or no follow-up. Concerning outpatient follow-up, psychotherapy seems to be the most suitable and effective treatment [26,32,41]. There are still several forms of psychotherapy (cognitive-behavioral, psychoeducational, individual, group, family) used in practice, to help patients manage their behavioral disorders and their social relationships [28,36]. The literature specifies that some psychotherapies are better adapted depending on the type of personality disorder [28]. However, current studies do not allow us to assert the superiority of one method over another [28,43]. Pharmacological treatment may also be instituted based on the symptomatology, or the potentially associated psychiatric diagnosis and therefore requires periodic follow-up with a psychiatrist. In some countries, specific structures are set up, favoring intensive outpatient monitoring, using a multidisciplinary approach [20,44]. The aim is to reduce the risk of hospitalization. However, according to an English study, 20.6% of patients with a personality disorder do not have outpatient or hospital follow-up after the diagnosis has been made [45], thus representing a larger percentage than patients with outpatient follow-up (19.2%) [45]. These studies prove that developing the already existing healthcare network is essential to improve the care of the patients concerned.

Along with this potential impact of comorbid personality disorders, several other factors that could potentially influence the decision during urgent forensic assessment were highlighted during our univariate analyses. Indeed, among demographic factors, female gender and being single were associated with higher risk of positive urgent forensic assessment. However, in the literature, the female gender is generally described as protective for involuntary psychiatric hospitalizations [9,46]. This difference from the literature could potentially be explained by the fact that in our sample, men had a higher prevalence of comorbid substance use disorders which more frequently promote induced psychiatric disorders that do not necessarily require long-term involuntary psychiatric hospitalizations [47]. Regarding being single, the results of this study are consistent with those in the literature in indicating that the absence of family structure is associated with higher risk of involuntary psychiatric hospitalizations following the potential absence of third parties who could provide a reliable alternative to the establishment of compulsory hospital care [9,46]. Furthermore, among the clinical factors, the existence of a psychiatric history (outpatient follow-up/hospitalization), the presence of at least one medical contact in the 3 months preceding the assessment, and the initiation of the assessment procedure by a health professional play a major role in the decision to approve the urgent forensic assessment. Indeed, these different factors seem to indicate that the decision of involuntary psychiatric hospitalization is taken as a last resort by psychiatrists carrying out urgent forensic assessments after the prior failure of other treatment alternatives [48]. Regarding psychiatric factors, the theoretical presence of neuroleptic and thymostabilizer medication in usual treatment is associated with higher risk of involuntary psychiatric hospitalizations in patients referred for urgent forensic assessment, which could be explained by the fact that these patients are frequently in therapeutic rupture characterized by a partial or complete cessation of their medication when they arrive with the police at the psychiatric emergency room [49]. Furthermore, consistent with the available data, the presence of some psychiatric diagnoses (bipolar and psychotic disorder) and some elements of dangerousness (suicidality and behavioral disorder) seem to have a central influence in the decision to confirm the urgent forensic assessment during the process of checking the legal criteria between psychiatrists [9,50,51]. Thus, based on these different elements, it is essential to carry out a holistic assessment of patients referred for urgent forensic assessment going beyond the three criteria of the law alone in order to allow the best possible decision regarding the implementation of potential involuntary psychiatric hospitalizations characterized by a partial or total suspension of some individual freedoms.

### 4.1. Limitations

The single-center retrospective design of this study introduces potential for selection bias and incomplete data. First of all, for some patients, several assessments were carried out during the period studied, some concluding with an involuntary psychiatric hospitalization, others not. However, only the first assessment of each patient having been taken into account, there is a potential loss of information which could lead to underestimating the number of involuntary psychiatric hospitalizations. Nevertheless, this choice to focus only on the first urgent forensic assessment imposed on patients was justified by our desire to avoid introducing additional bias into this study. Indeed, the repetition of urgent forensic assessment may reflect a progressive exhaustion of voluntary care alternatives for the patients concerned, which could promote a bias in the interpretation of the risk of involuntary psychiatric hospitalizations obtained. In addition, the emergency files used to collect data were not always complete. The psychiatric state of the patient, agitation, or on the contrary muteness, language barrier, the impossibility of obtaining a quality hetero-anamnesis are elements that may lead to a loss of information, particularly concerning psychiatric history and other psychiatric diagnoses previously made. In addition, the retrospective design makes it impossible to find additional elements at the time of encoding. However, since the urgent forensic assessments included for this study were all carried out in the context of a procedure requested by the King’s Prosecutor, the number of assessments excluded due to missing data was very limited (14), which could be explained by the fact that this judicial context requires the best possible data collection to enable the potential decision-making regarding involuntary psychiatric hospitalizations. This limited exclusion of urgent forensic assessments due to missing data should therefore not have significantly impacted the results obtained in this study. On the other hand, the diagnosis of personality disorder seems relatively infrequent (14.2%). Personality disorder is described in the literature as frequently underdiagnosed, despite the fact that it is frequently associated with a mood disorder [52,53]. Indeed, a considerable amount of our psychological information, such as somatic symptoms and personality evaluations, was obtained through self-reported assessments, potentially leading to biased self-representation [54]. Finally, although each decision concerning the outcome of urgent forensic assessment is always taken after a discussion between the junior psychiatrist present in the emergency room and a senior psychiatrist experienced in carrying out forensic assessments to limit as much as possible any risk of subjectivity in the interpretation of legal criteria, the fact that not all the assessments included in this study were carried out by the same psychiatrist may possibly introduce a bias in the interpretation of the results of this study.

### 4.2. Perspectives

Our results highlight the importance of further research on this topic. It would be interesting to explore the issue further with prospective, multicenter studies and large cohorts from all regions of Belgium in order to identify more precise results. In addition, in these future studies, the implementation of a standardized formula for carrying out urgent forensic assessment could help to limit the risk of subjectivity when interpreting the legal criteria for involuntary psychiatric hospitalizations. Furthermore, it also seems important to be able to compare the results obtained in Belgium with other countries to assess the potential impact of national legislation on the risk of involuntary psychiatric hospitalizations associated with comorbid personality disorders. Although the impact of some comorbid personality disorder subtypes was investigated in this study, it seems important to be able to replicate this study on a larger sample to determine the potential impact of other less frequent personality subtypes on the risk of involuntary psychiatric hospitalizations. The saturation of constrained care networks in Belgium requires a better understanding of the factors leading a patient to need involuntary psychiatric hospitalization in order to strengthen upstream care and identify the factors which could slow down access to timely and effective care.

## 5. Conclusions

The objective of our study was to determine the impact of comorbid personality disorders on the risk of involuntary psychiatric hospitalization. The results of our study indicate that comorbid personality disorders are associated with lower risk of involuntary psychiatric hospitalization in the current organization of health care in Belgium. This study was important in trying to limit the use of involuntary psychiatric hospitalization to justified situations, but additional research is necessary in order to clarify our results and to be able to generalize them to a larger population.

## Data Availability

The data presented in this study are available on request from the corresponding author due to internal institutional procedures.
